# Supplementary material for: The influence of 4-thiouridine labeling on pre-mRNA splicing outcomes
Source: PLoS One. 2021 Dec 13;16(12):e0257503. doi: 10.1371/journal.pone.0257503 (PMC8668116; doi:10.1371/journal.pone.0257503)
Supplement: S1 Raw images — (PDF) [file pone.0257503.s002.pdf]

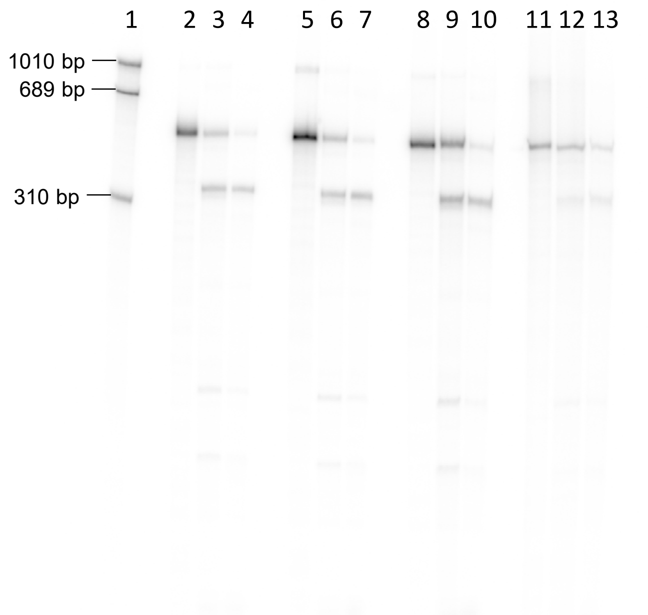

***In vitro* pre-mRNA splicing of β-Globin (gel from Fig.1B)**

Imaged with GE Typhoon Imager

1: RNA Ladder
2: 0% 4sU 0min
3: 0% 4sU 1hr
4: 0% 4sU 2hr
5: 2.5% 4sU 0min
6: 2.5% 4sU 1hr
7: 2.5% 4sU 2hr
8: 30% 4sU 0min
9: 30% 4sU 1hr
10: 30% 4sU 2hr
11: 100% 4sU 0min
12: 100% 4sU 1hr
13: 100% 4sU 2hr

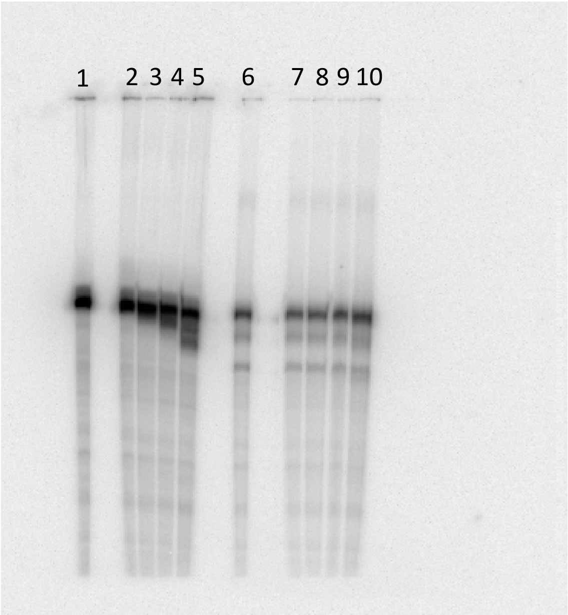

***In vitro* degradation of AdML (gel from Fig. 2A)**

Imaged with GE Typhoon Imager

1: 0% 4sU 0min
2: 0% 4sU 5min
3: 0% 4sU 15min
4: 0% 4sU 30 min
5: 0% 4sU 90 min
6: 100% 4sU 0min
7: 100% 4sU 5min
8: 100% 4sU 15min
9: 100% 4sU 30min
10: 100% 4sU 90min

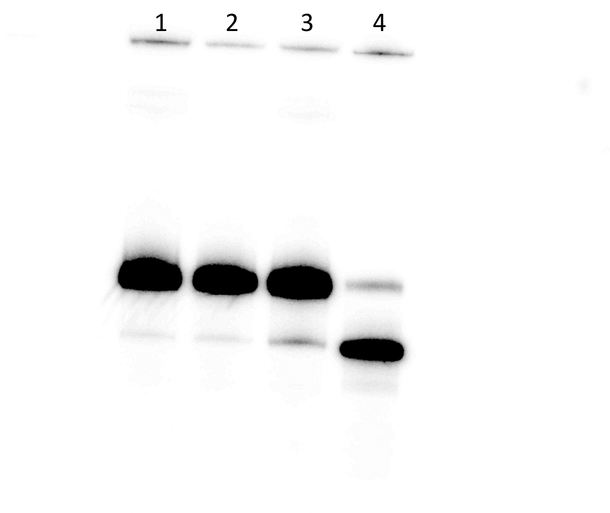

***In vitro* transcription of AdML (gel from Fig. 3A)**

Autoradiogram

1: 0% 4sU pre-mRNA
2: 2.5% 4sU pre-mRNA
3: 30% 4sU pre-mRNA
4: 100% 4sU pre-mRNA

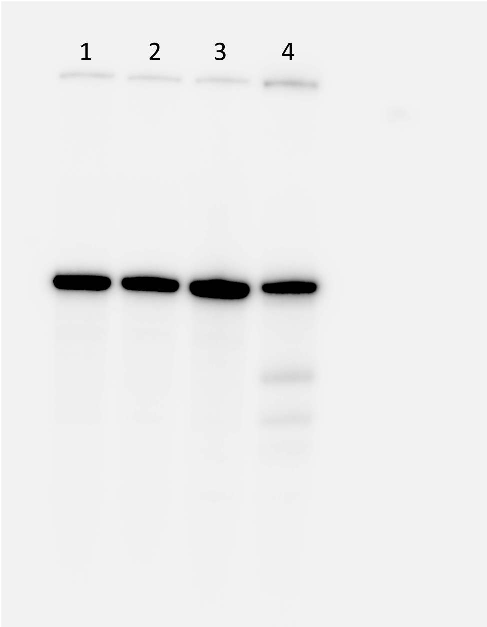

***In vitro* transcription of β-Globin (gel from Fig. 3B)**

Autoradiogram

1: 0% 4sU pre-mRNA
2: 2.5% 4sU pre-mRNA
3: 30% 4sU pre-mRNA
4: 100% 4sU pre-mRNA

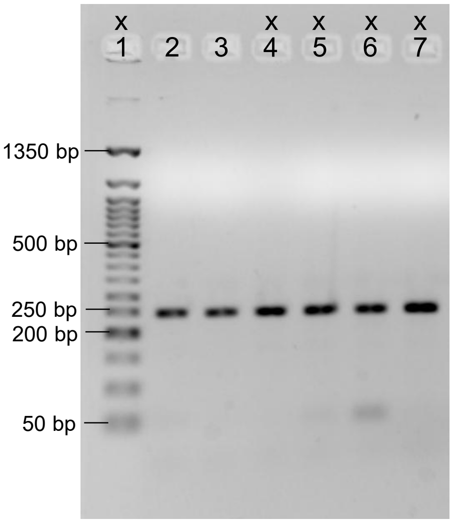

2hr *ADAP2* (Exons 6-8) RT-PCR (Fig. 4A)

Imaged with Bio-Rad Gel Doc

1: 50bp DNA Ladder
2: No 4sU #1
3: 40μM 4sU #1
4: No 4sU #2
5: 40μM 4sU #2
6: No 4sU #3
7: 40μM 4sU #3

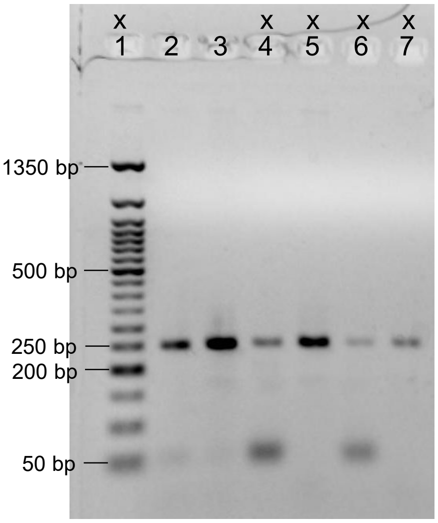

24hr *ADAP2* (Exons 6-8) RT-PCR (Fig. 4A)

Imaged with Bio-Rad Gel Doc

1: 50bp DNA Ladder
2: No 4sU #1
3: 40μM 4sU #1
4: No 4sU #2
5: 40μM 4sU #2
6: No 4sU #3
7: 40μM 4sU #3

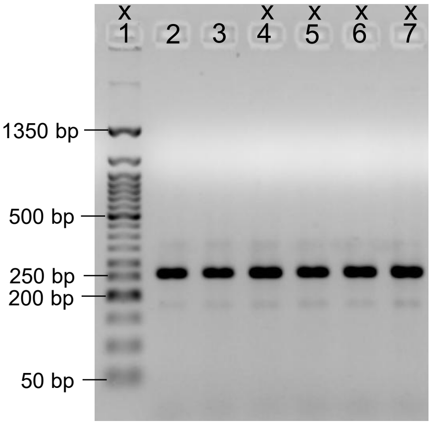

2hr *DOLPP1* (Exons 2-5) RT-PCR (Fig. 4A)

Imaged with Bio-Rad Gel Doc

1: 50bp DNA Ladder
2: No 4sU #1
3: 40μM 4sU #1
4: No 4sU #2
5: 40μM 4sU #2
6: No 4sU #3
7: 40μM 4sU #3

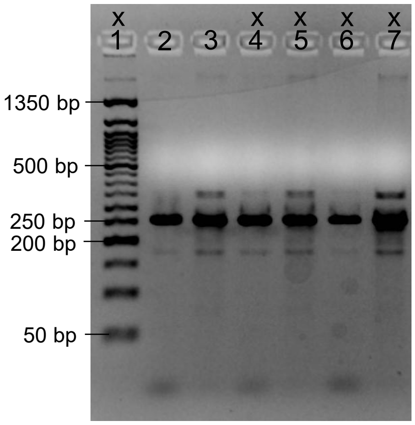

24hr *DOLPP1* (Exons 2-5) RT-PCR (Fig. 4A)

Imaged with Bio-Rad Gel Doc

1: 50bp DNA Ladder
2: No 4sU #1
3: 40μM 4sU #1
4: No 4sU #2
5: 40μM 4sU #2
6: No 4sU #3
7: 40μM 4sU #3

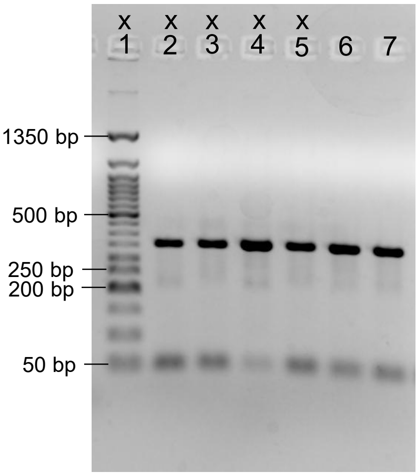

2hr *ZNF711* (Exons 4-6) RT-PCR (Fig. 4A)

Imaged with Bio-Rad Gel Doc

1: 50bp DNA Ladder
2: No 4sU #1
3: 40μM 4sU #1
4: No 4sU #2
5: 40μM 4sU #2
6: No 4sU #3
7: 40μM 4sU #3

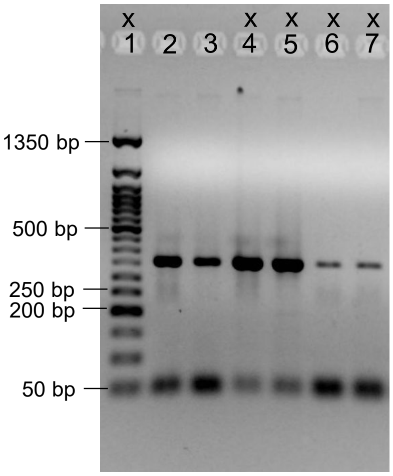

24hr *ZNF711* (Exons 4-6) RT-PCR (Fig. 4A)

Imaged with Bio-Rad Gel Doc

1: 50bp DNA Ladder
2: No 4sU #1
3: 40μM 4sU #1
4: No 4sU #2
5: 40μM 4sU #2
6: No 4sU #3
7: 40μM 4sU #3

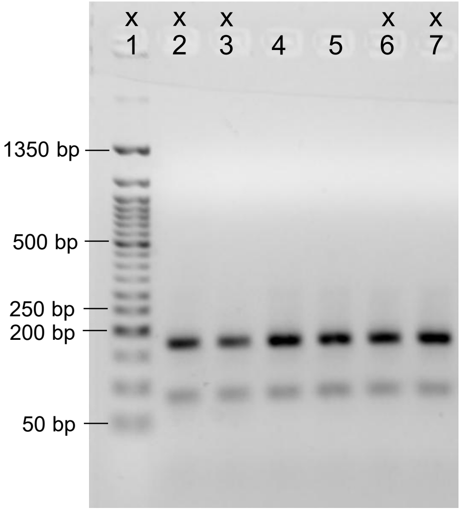

2hr *ADAP2* (Exons 2-4) RT-PCR (Fig. 4B)

Imaged with Bio-Rad Gel Doc

1: 50bp DNA Ladder
2: No 4sU #1
3: 40μM 4sU #1
4: No 4sU #2
5: 40μM 4sU #2
6: No 4sU #3
7: 40μM 4sU #3

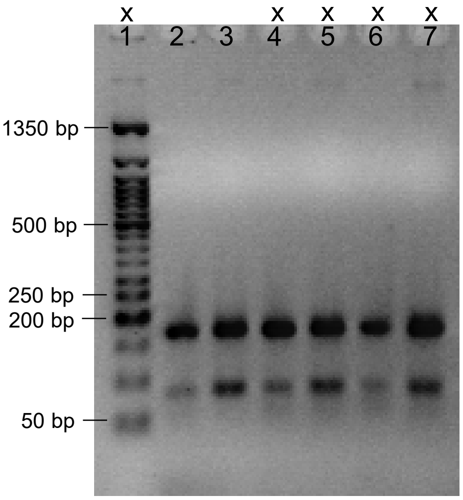

24hr *ADAP2* (Exons 2-4) RT-PCR (Fig. 4B)

Imaged with Bio-Rad Gel Doc

1: 50bp DNA Ladder
2: No 4sU #1
3: 40μM 4sU #1
4: No 4sU #2
5: 40μM 4sU #2
6: No 4sU #3
7: 40μM 4sU #3

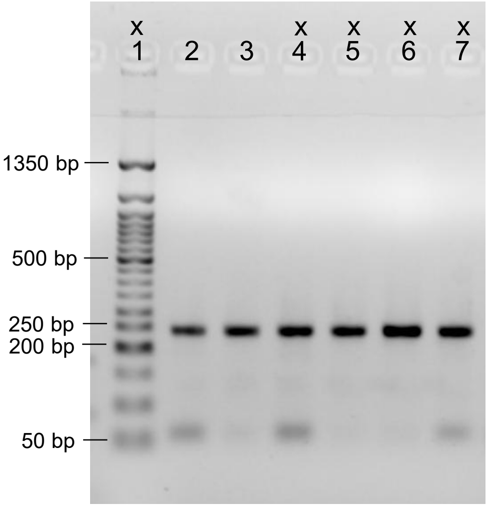

2hr *DOLPP1* (Exons 5-7) RT-PCR (Fig. 4B)

Imaged with Bio-Rad Gel Doc

1: 50bp DNA Ladder
2: No 4sU #1
3: 40μM 4sU #1
4: No 4sU #2
5: 40μM 4sU #2
6: No 4sU #3
7: 40μM 4sU #3

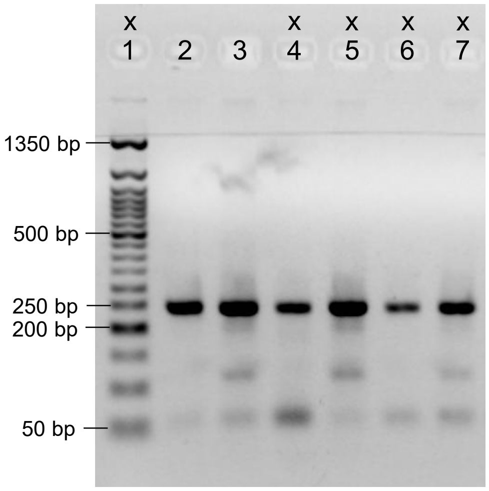

24hr *DOLPP1* (Exons 5-7) RT-PCR (Fig. 4B)

Imaged with Bio-Rad Gel Doc

1: 50bp DNA Ladder
2: No 4sU #1
3: 40μM 4sU #1
4: No 4sU #2
5: 40μM 4sU #2
6: No 4sU #3
7: 40μM 4sU #3

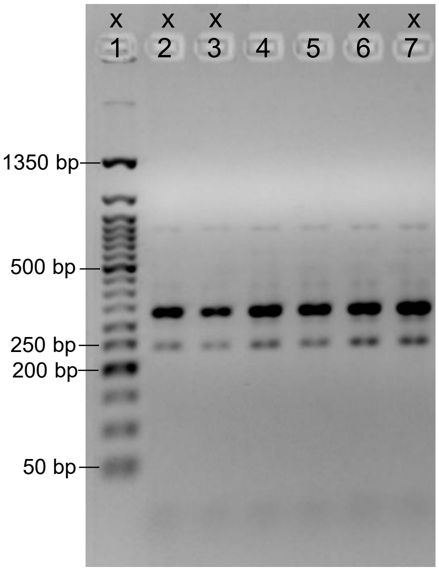

2hr *CLK2* (Exons 3-6) RT-PCR (Fig. 4B)

Imaged with Bio-Rad Gel Doc

1: 50bp DNA Ladder
2: No 4sU #1
3: 40μM 4sU #1
4: No 4sU #2
5: 40μM 4sU #2
6: No 4sU #3
7: 40μM 4sU #3

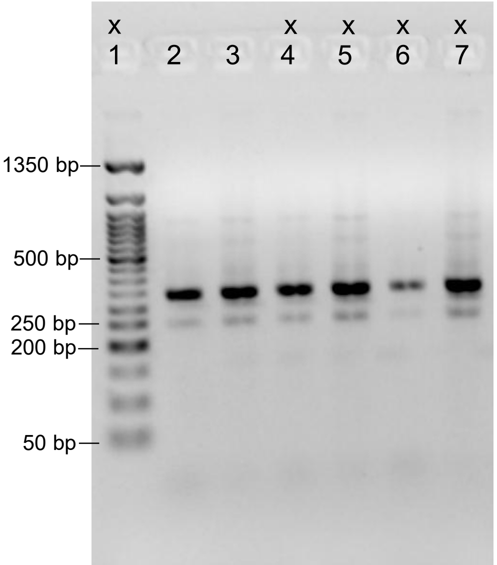

24hr *CLK2* (Exons 3-6) RT-PCR (Fig. 4B)

Imaged with Bio-Rad Gel Doc

1: 50bp DNA Ladder
2: No 4sU #1
3: 40μM 4sU #1
4: No 4sU #2
5: 40μM 4sU #2
6: No 4sU #3
7: 40μM 4sU #3

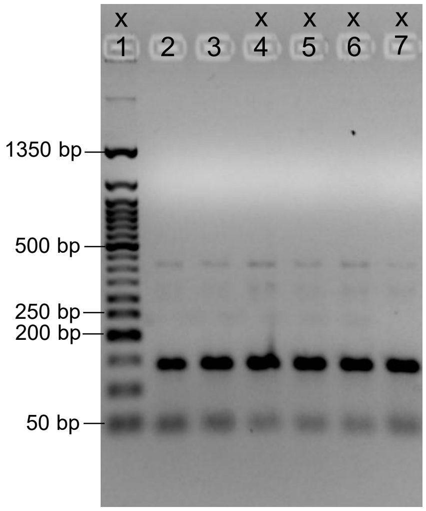

2hr *TRA2B* (Exons 1-3) RT-PCR (Fig. 4B)

Imaged with Bio-Rad Gel Doc

1: 50bp DNA Ladder
2: No 4sU #1
3: 40µM 4sU #1
4: No 4sU #2
5: 40µM 4sU #2
6: No 4sU #3
7: 40µM 4sU #3

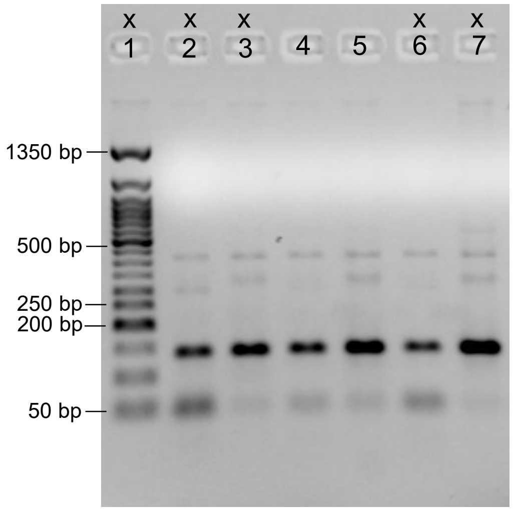

24hr *TRA2B* (Exons 1-3) RT-PCR (Fig. 4B)

Imaged with Bio-Rad Gel Doc

1: 50bp DNA Ladder
2: No 4sU #1
3: 40µM 4sU #1
4: No 4sU #2
5: 40µM 4sU #2
6: No 4sU #3
7: 40µM 4sU #3

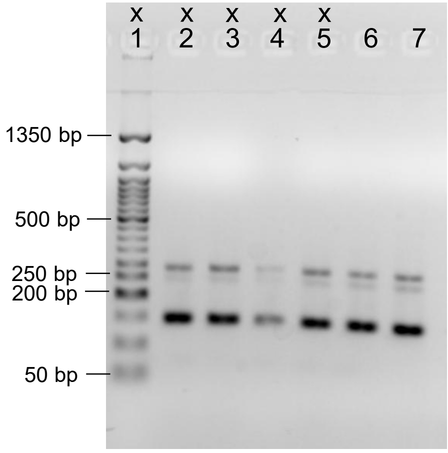

2hr *ZNF711* (Exons 6-8) RT-PCR (Fig. 4B)

Imaged with Bio-Rad Gel Doc

1: 50bp DNA Ladder
2: No 4sU #1
3: 40µM 4sU #1
4: No 4sU #2
5: 40µM 4sU #2
6: No 4sU #3
7: 40µM 4sU #3

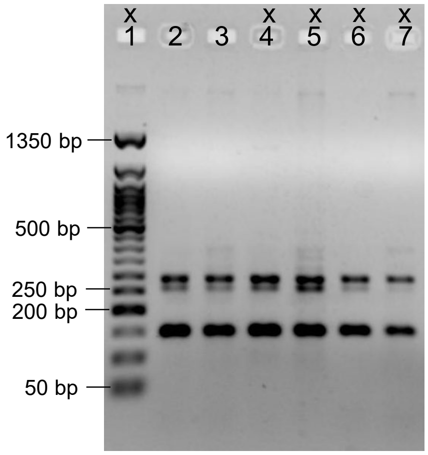

24hr *ZNF711* (Exons 6-8) RT-PCR (Fig. 4B)

Imaged with Bio-Rad Gel Doc

1: 50bp DNA Ladder
2: No 4sU #1
3: 40µM 4sU #1
4: No 4sU #2
5: 40µM 4sU #2
6: No 4sU #3
7: 40µM 4sU #3

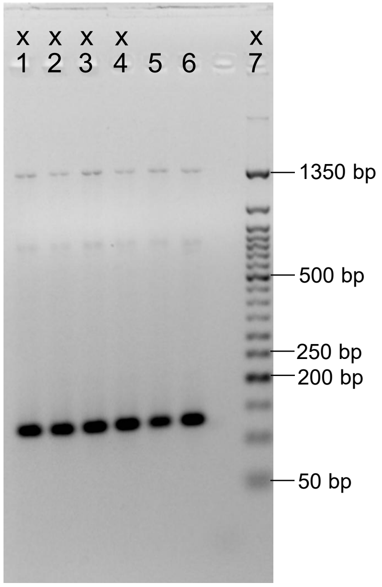

2hr *DDX5* (Exons 11-12) RT-PCR (Fig. 5B)
Imaged with Bio-Rad Gel Doc
1: No 4sU #1
2: 40μM 4sU #1
3: No 4sU #2
4: 40μM 4sU #2
5: No 4sU #3
6: 40μM 4sU #3
7: 50bp DNA Ladder

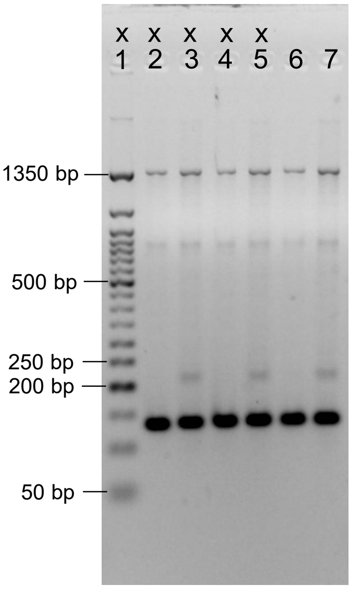

24hr *DDX5* (Exons 11-12) RT-PCR (Fig. 5B)
Imaged with Bio-Rad Gel Doc
1: 50bp DNA Ladder
2: No 4sU #1
3: 40μM 4sU #1
4: No 4sU #2
5: 40μM 4sU #2
6: No 4sU #3
7: 40μM 4sU #3

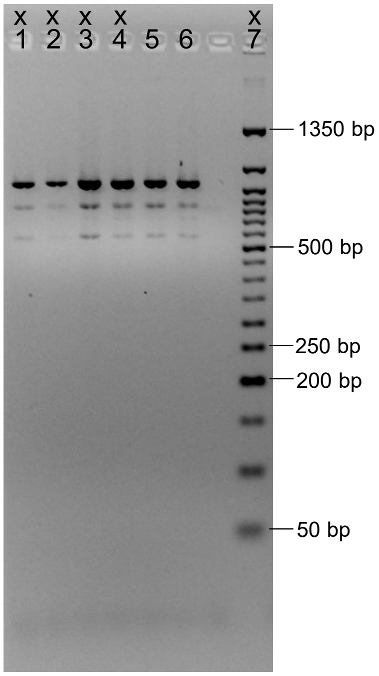

2hr *RIOK3* (Exons 5-10) RT-PCR (Fig. 5A)
Imaged with Bio-Rad Gel Doc
1: No 4sU #1
2: 40μM 4sU #1
3: No 4sU #2
4: 40μM 4sU #2
5: No 4sU #3
6: 40μM 4sU #3
7: 50bp DNA Ladder

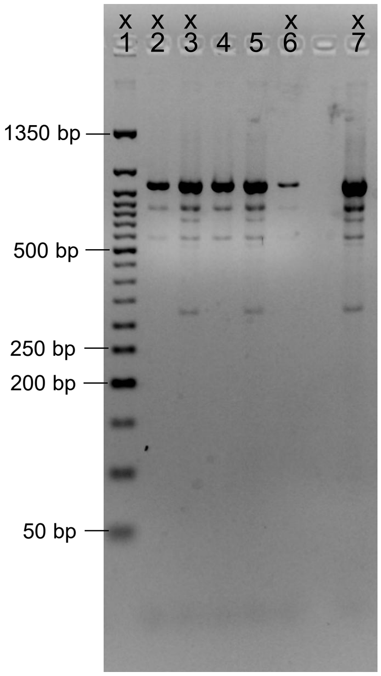

24hr *RIOK3* (Exons 5-10) RT-PCR (Fig. 5A)
Imaged with Bio-Rad Gel Doc
1: 50bp DNA Ladder
2: No 4sU #1
3: 40μM 4sU #1
4: No 4sU #2
5: 40μM 4sU #2
6: No 4sU #3
7: 40μM 4sU #3
