# Supplementary material for: The influence of 4-thiouridine labeling on pre-mRNA splicing outcomes
Source: PLoS One. 2021 Dec 13;16(12):e0257503. doi: 10.1371/journal.pone.0257503 (PMC8668116; doi:10.1371/journal.pone.0257503)
Supplement: Attachment — Submitted filename: Review.pdf [file pone.0257503.s003.pdf]

The manuscript “The influence of 4-thiouridine labeling on pre-mRNA splicing outcomes” by Altieri and Hertel describes how the commonly used 4SU modified base has limited effect on splicing in cells at the concentrations employed for most experiments. This result should give other researchers confidence that use of 4SU should not strongly bias their experimental outcomes for constitutive splicing. However, it also is a reminder that the splicing events most responsive to regulation, are also most likely to be sensitive to experimental conditions. The authors also examine in vitro RNA splicing and find some changes in splicing efficiency and stability with very high incorporation of 4SU. Overall, the data are clearly presented, and their conclusions are generally well supported. The authors should address the following minor points to improve the presentation of the studies.

1- For in vitro splicing assays, the methods should explicitly describe the calculation to determine splicing efficiency, which is typically band intensity of the product mRNA divided by the summed band intensity for pre-mRNA, one of the 1st intermediate products, and mRNA- each corrected for background and the number of radiolabeled nucleotides incorporated in each species.

2-Changes in in vitro splicing efficiency can also be due to differential stability of the RNA species. Indeed, the data shown in Fig. 1B indicates that the pre-mRNA is apparently disappearing at higher rate than mRNA formation. The authors also show that high 4SU incorporation stabilizes the pre-mRNA for the AdML transcript. Is it possible that the calculated decrease in splicing with high 4SU may instead reflect increased pre-mRNA stability? Discussion of this potentially confounding issue is needed.

3-What type of variation is seen with degradation assay shown if Figure 2?

4- The nature of experimental replicates for Figures 4 and 5 should be stated. Are they biological replicates of RNA extracted from cells or technical replicates of RT-PCR analysis? The former would better support the conclusions related to 4SU induced splicing changes for the same reason that they are examining sensitive splicing events.

5- Is there a possibility that 4SU induces aberrant splicing? There are some new bands that appear at 24 hr with RSU appearing with DOLP11, TRA2B and DDX5.
