# Supplementary material for: The influence of 4-thiouridine labeling on pre-mRNA splicing outcomes
Source: PLoS One. 2021 Dec 13;16(12):e0257503. doi: 10.1371/journal.pone.0257503 (PMC8668116; doi:10.1371/journal.pone.0257503)
Supplement: Attachment — Submitted filename: Response to Reviewers.docx [file pone.0257503.s004.docx]

We thank the reviewers for their thorough and careful reading of the manuscript. We have addressed their comments and concerns to the best of our abilities. We have made the necessary changes to the manuscript per the reviewers’ feedback, and we have addressed the questions raised in our responses. The reviewer’s critiques are listed in *italic* first and our responses are headed by the word “Response”:

**Reviewer #1:**

*The presented study is significant, since until now consequences of 4sU labeling on nascent pre-mRNA splicing have not been evaluated. The results of the study indicate that pre-mRNA splicing can be impacted by the incorporation of 4sU into pre-mRNA. The effect was particularly pronounced in in vitro experiments (splicing, transcription, and pre-mRNA stability) at elevated levels of 4sU incorporation. In addition, how much 4sU influenced splicing depended on the strength of the splice sites in question. Overall, the results showed that in case of splicing, the incorporation of 4sU into pre-mRNAs is not without consequences and can affect pre-mRNA splicing. The consequences become detectable at elevated levels of 4sU incorporation and in the context of suboptimal splice sites. Therefore, the study suggests that appropriate controls should be included when metabolic labeling is used.

1. Check for mistakes. For example,
(a) in “Materials and Methods” it is stated that in in vitro transcription the relative amount of 4sU was 0%, 2.5%, 15%, 30%, 100%. However, in the Results “15%” sample is absent.*

Response:

We thank the reviewer for his/her positive feedback on our work and their careful reading of our manuscript. We are sorry for the oversight mistake. In the revised manuscript we have fixed this error.

*(b) in “Material and Methods”, page 6, “HEK293 metabolic labeling” there should be a space between “protocol was followed” and “[6]” (line 134).*

Response:

We are sorry for the oversight mistake. In the revised manuscript we have fixed this error.

*(c) In “Results”, line 231, a bracket is missing in front of “A”*

Response:

We are sorry for the oversight mistake. In the revised manuscript we have fixed this error.

*2. Why write “In vitro splicing was carried out …for 90 min …” if the time points were “1 hour” and “2 hours” and there is no “90 min” time point at all (in “Materials and Methods”).*

Response:

Thank you for pointing out this mistake. In the revised manuscript we have fixed this error.

*3. In Figure 1A, please, mark the 3’ and 5’ splice sites in addition to just indicating their strength.*

Response:

The revised Figure 1 now defines the 3’ and 5’ splice sites and their strength.

*4. Is there any explanation to why the total signal is dramatically weaker at “1 hour” as compared to “t=0” input RNA specifically at 0% and 2.5% labeling (in Figure 1B)? Also, why the pre-mRNA band at t=0 is significantly weaker at 100% 4sU incorporation as compared to 0%, 2.5% and 30%? If different amount of pre-mRNA was used for in vitro splicing, please, specify in “Materials and Methods”.*

Response:

At optimized *in vitro* splicing conditions (energy source, energy regeneration source, MgCl2, DTT) T7 polymerase-generated pre-mRNA (only cap analog, no poly A tail) is attacked and degraded by RNases contained within nuclear extract. This happens within the first ~15 minutes of the splicing reaction as spliceosomal components and RNases compete for the pre-mRNA that has been added to the reaction. RNAs that associate with the spliceosome are protected from this degradation. In a typical splicing reaction ~50-75% of the input RNA is degraded quickly. This is a well-documented phenomenon that is often countered by loading different amounts of the splicing reaction onto the gel – with the result that time courses do not show the visual loss of RNA intensity. As splicing efficiencies at various time points are calculated based on the relative representation of pre-mRNA and mRNA, loading only a fraction of a time point will not alter the calculated splicing efficiency. We have opted not to load fractions of time points, but the entire reaction for each time point. As a result, our *in vitro* splicing gels show differential total counts in each of the time point lanes. In the revised manuscript, we state our loading approach in the ‘Materials and Methods’.

*5. Why “24 h” ZNF711 signal is so weak in the presence of 4sU?*

Response:

We thank the reviewer for pointing out this anomaly, which was caused by a loading error. We have re-run the experiments and now show an updated RT-PCR image. The corresponding quantitation was updated accordingly.

*6. In Figure 4B, please, provide quantitation for ADAP2 similarly as you did for DOLPP1 since visually (gel image) 4sU labelling at 24 hr appears to cause a difference in ADAP2 splicing pattern. Maybe even provide quantitation for all five exon skipping events.*

Response:

As suggested, in the revised manuscript all images showing RT-PCR results are now accompanied with quantitation.

**Reviewer #2:**

*The manuscript “The influence of 4-thiouridine labeling on pre-mRNA splicing outcomes” by Altieri and Hertel describes how the commonly used 4SU modified base has limited effect on splicing in cells at the concentrations employed for most experiments. This result should give other researchers confidence that use of 4SU should not strongly bias their experimental outcomes for constitutive splicing. However, it also is a reminder that the splicing events most responsive to regulation, are also most likely to be sensitive to experimental conditions. The authors also examine in vitro RNA splicing and find some changes in splicing efficiency and stability with very high incorporation of 4SU. Overall, the data are clearly presented, and their conclusions are generally well supported. The authors should address the following minor points to improve the presentation of the studies.

1- For in vitro splicing assays, the methods should explicitly describe the calculation to determine splicing efficiency, which is typically band intensity of the product mRNA divided by the summed band intensity for pre-mRNA, one of the 1st intermediate products, and mRNA- each corrected for background and the number of radiolabeled nucleotides incorporated in each species.*

Response:

As suggested, the revised manuscript now contains a description how the efficiency of splicing was calculated. This information is included in the ‘Materials & Methods’ section.

*2-Changes in in vitro splicing efficiency can also be due to differential stability of the RNA species. Indeed, the data shown in Fig. 1B indicates that the pre-mRNA is apparently disappearing at higher rate than mRNA formation. The authors also show that high 4SU incorporation stabilizes the pre-mRNA for the AdML transcript. Is it possible that the calculated decrease in splicing with high 4SU may instead reflect increased pre-mRNA stability? Discussion of this potentially confounding issue is needed.*

Response:

The reviewer pointed out a plausible alternative explanation for the changes in splicing efficiency, namely that differential degradation rates of the pre-mRNA might influence calculated splicing efficiencies. To understand the influence of degradation differences on splicing differences it is best to evaluate the profiles of our β-globin substrate. It shows the most striking splicing differences when comparing fully and not modified RNA substrates (Fig. 1B,C). However, as stated in the manuscript, we did not detect statistically significant differences in the stability of the 0% and 100% 4sU modified pre-mRNAs. Thus, for the β-globin substrate the difference in splicing is not influenced by stability differences – it is all splicing. A similar analysis of the compounding splicing/degradation factors for the AdML substrate shows minimal, but not statistically significant splice differences (Fig. 1D), but stability differences (Fig. 2). Thus, it is possible that the trend of reduced splicing efficiency observed for the AdML constructs (although not statistically significant) could be a consequence of stability differences. So, for AdML, these potentially confounding effects cannot be uncoupled, as hinted to by the reviewer. In the revised manuscript now discuss the potential of these confounding factors.

*3-What type of variation is seen with degradation assay shown if Figure 2?*

Response:

We apologize for not showing the variation of the data shown in Figure 2. This has been corrected and the revised figure now shows that variation with error bars. We thank the reviewer for pointing out this oversight.

*4- The nature of experimental replicates for Figures 4 and 5 should be stated. Are they biological replicates of RNA extracted from cells or technical replicates of RT-PCR analysis? The former would better support the conclusions related to 4SU induced splicing changes for the same reason that they are examining sensitive splicing events.*

Response:

All replicates are biological replicates, and the revised manuscript now explicitly states this.

*5- Is there a possibility that 4SU induces aberrant splicing? There are some new bands that appear at 24 hr with 4SU appearing with DOLP11, TRA2B and DDX5.*

Response:

Yes, it is possible that 4sU induces aberrant splicing. The reviewer is correct in pointing out that additional RT-PCR bands appear in the 24hrs time point for the genes listed. Without sequencing the PCR product, it is difficult to assign identities to these bands, meaning assigning whether the bands represent aberrant splicing. In the revised manuscript we now point out the additional bands as possible, but unconfirmed evidence of aberrant splicing.
