# Supplementary material for: The influence of 4-thiouridine labeling on pre-mRNA splicing outcomes
Source: PLoS One. 2021 Dec 13;16(12):e0257503. doi: 10.1371/journal.pone.0257503 (PMC8668116; doi:10.1371/journal.pone.0257503)
Supplement: Peer review history

# Peer review: Decision Letter 0

**DOI:** 10.1371/journal.pone.0257503.r001

**Authors:** Ravindra N Singh

4 Oct 2021

PONE-D-21-28397

The influence of 4-thiouridine labeling on pre-mRNA splicing outcomes

PLOS ONE

Dear Dr. Hertel,

Thank you for submitting your manuscript to PLOS ONE. After careful consideration, we feel that it has merit but does not fully meet PLOS ONE’s publication criteria as it currently stands. Therefore, we invite you to submit a revised version of the manuscript that addresses the points raised during the review process.

Both reviewers liked your study. However, they had minor concerns that could be addressed without any new experiments. Please be as detailed as possible in your explanations. 

Please submit your revised manuscript by Nov 18 2021 11:59PM. If you will need more time than this to complete your revisions, please reply to this message or contact the journal office at plosone@plos.org. When you're ready to submit your revision, log on to https://www.editorialmanager.com/pone/ and select the 'Submissions Needing Revision' folder to locate your manuscript file.

Please include the following items when submitting your revised manuscript:

- A rebuttal letter that responds to each point raised by the academic editor and reviewer(s). You should upload this letter as a separate file labeled 'Response to Reviewers'.
- A marked-up copy of your manuscript that highlights changes made to the original version. You should upload this as a separate file labeled 'Revised Manuscript with Track Changes'.
- An unmarked version of your revised paper without tracked changes. You should upload this as a separate file labeled 'Manuscript'.

If you would like to make changes to your financial disclosure, please include your updated statement in your cover letter. Guidelines for resubmitting your figure files are available below the reviewer comments at the end of this letter.

If applicable, we recommend that you deposit your laboratory protocols in protocols.io to enhance the reproducibility of your results. Protocols.io assigns your protocol its own identifier (DOI) so that it can be cited independently in the future. For instructions see: https://journals.plos.org/plosone/s/submission-guidelines#loc-laboratory-protocols. Additionally, PLOS ONE offers an option for publishing peer-reviewed Lab Protocol articles, which describe protocols hosted on protocols.io. Read more information on sharing protocols at https://plos.org/protocols?utm_medium=editorial-email&utm_source=authorletters&utm_campaign=protocols.

We look forward to receiving your revised manuscript.

Kind regards,

Ravindra N Singh, Ph.D.

Academic Editor

PLOS ONE

Journal Requirements:

When submitting your revision, we need you to address these additional requirements.

1. Please ensure that your manuscript meets PLOS ONE's style requirements, including those for file naming. The PLOS ONE style templates can be found at 

https://journals.plos.org/plosone/s/file?id=wjVg/PLOSOne_formatting_sample_main_body.pdf and 

https://journals.plos.org/plosone/s/file?id=ba62/PLOSOne_formatting_sample_title_authors_affiliations.pdf.

2. Please review your reference list to ensure that it is complete and correct. If you have cited papers that have been retracted, please include the rationale for doing so in the manuscript text, or remove these references and replace them with relevant current references. Any changes to the reference list should be mentioned in the rebuttal letter that accompanies your revised manuscript. If you need to cite a retracted article, indicate the article’s retracted status in the References list and also include a citation and full reference for the retraction notice.

3. We note that the grant information you provided in the ‘Funding Information’ and ‘Financial Disclosure’ sections do not match. 

When you resubmit, please ensure that you provide the correct grant numbers for the awards you received for your study in the ‘Funding Information’ section.

4. In your Data Availability statement, you have not specified where the minimal data set underlying the results described in your manuscript can be found. PLOS defines a study's minimal data set as the underlying data used to reach the conclusions drawn in the manuscript and any additional data required to replicate the reported study findings in their entirety. All PLOS journals require that the minimal data set be made fully available. For more information about our data policy, please see http://journals.plos.org/plosone/s/data-availability.

Upon re-submitting your revised manuscript, please upload your study’s minimal underlying data set as either Supporting Information files or to a stable, public repository and include the relevant URLs, DOIs, or accession numbers within your revised cover letter. For a list of acceptable repositories, please see http://journals.plos.org/plosone/s/data-availability#loc-recommended-repositories. Any potentially identifying patient information must be fully anonymized.

Important: If there are ethical or legal restrictions to sharing your data publicly, please explain these restrictions in detail. Please see our guidelines for more information on what we consider unacceptable restrictions to publicly sharing data: http://journals.plos.org/plosone/s/data-availability#loc-unacceptable-data-access-restrictions. Note that it is not acceptable for the authors to be the sole named individuals responsible for ensuring data access.

We will update your Data Availability statement to reflect the information you provide in your cover letter.

5. PLOS ONE now requires that authors provide the original uncropped and unadjusted images underlying all blot or gel results reported in a submission’s figures or Supporting Information files. This policy and the journal’s other requirements for blot/gel reporting and figure preparation are described in detail at https://journals.plos.org/plosone/s/figures#loc-blot-and-gel-reporting-requirements and https://journals.plos.org/plosone/s/figures#loc-preparing-figures-from-image-files. When you submit your revised manuscript, please ensure that your figures adhere fully to these guidelines and provide the original underlying images for all blot or gel data reported in your submission. See the following link for instructions on providing the original image data: https://journals.plos.org/plosone/s/figures#loc-original-images-for-blots-and-gels. 

  

In your cover letter, please note whether your blot/gel image data are in Supporting Information or posted at a public data repository, provide the repository URL if relevant, and provide specific details as to which raw blot/gel images, if any, are not available. Email us at plosone@plos.org if you have any questions.

6. Please upload a new copy of Figure 5 as the detail is not clear. Please follow the link for more information: " ext-link-type="uri" xlink:type="simple">https://blogs.plos.org/plos/2019/06/looking-good-tips-for-creating-your-plos-figures-graphics/" " ext-link-type="uri" xlink:type="simple">https://blogs.plos.org/plos/2019/06/looking-good-tips-for-creating-your-plos-figures-graphics/"

[Note: HTML markup is below. Please do not edit.]

Reviewers' comments:

Reviewer's Responses to Questions

**Comments to the Author**

1. Is the manuscript technically sound, and do the data support the conclusions?

The manuscript must describe a technically sound piece of scientific research with data that supports the conclusions. Experiments must have been conducted rigorously, with appropriate controls, replication, and sample sizes. The conclusions must be drawn appropriately based on the data presented. 

Reviewer #1: Yes

Reviewer #2: Yes

********** 

2. Has the statistical analysis been performed appropriately and rigorously? 

Reviewer #1: Yes

Reviewer #2: Yes

********** 

3. Have the authors made all data underlying the findings in their manuscript fully available?

The PLOS Data policy requires authors to make all data underlying the findings described in their manuscript fully available without restriction, with rare exception (please refer to the Data Availability Statement in the manuscript PDF file). The data should be provided as part of the manuscript or its supporting information, or deposited to a public repository. For example, in addition to summary statistics, the data points behind means, medians and variance measures should be available. If there are restrictions on publicly sharing data—e.g. participant privacy or use of data from a third party—those must be specified.

Reviewer #1: Yes

Reviewer #2: Yes

********** 

4. Is the manuscript presented in an intelligible fashion and written in standard English?

PLOS ONE does not copyedit accepted manuscripts, so the language in submitted articles must be clear, correct, and unambiguous. Any typographical or grammatical errors should be corrected at revision, so please note any specific errors here.

Reviewer #1: Yes

Reviewer #2: Yes

********** 

5. Review Comments to the Author

Please use the space provided to explain your answers to the questions above. You may also include additional comments for the author, including concerns about dual publication, research ethics, or publication ethics. (Please upload your review as an attachment if it exceeds 20,000 characters)

Reviewer #1: In this manuscript the authors tested whether metabolic labeling with 4-thiouridine (4sU), a tool commonly used to investigate different aspects of RNA metabolism, creates any biases/negative effect on pre-mRNA splicing. To do so, they performed in vitro experiments with well controlled levels of 4sU incorporation (from 0 to 100%) as well as cell culture experiments with commonly used 4sU concentrations.

The presented study is significant, since until now consequences of 4sU labeling on nascent pre-mRNA splicing have not been evaluated. The results of the study indicate that pre-mRNA splicing can be impacted by the incorporation of 4sU into pre-mRNA. The effect was particularly pronounced in in vitro experiments (splicing, transcription, and pre-mRNA stability) at elevated levels of 4sU incorporation. In addition, how much 4sU influenced splicing depended on the strength of the splice sites in question. Overall, the results showed that in case of splicing, the incorporation of 4sU into pre-mRNAs is not without consequences and can affect pre-mRNA splicing. The consequences become detectable at elevated levels of 4sU incorporation and in the context of suboptimal splice sites. Therefore, the study suggests that appropriate controls should be included when metabolic labeling is used.

1. Check for mistakes. For example,

(a) in “Materials and Methods” it is stated that in in vitro transcription the relative amount of 4sU was 0%, 2.5%, 15%, 30%, 100%. However, in the Results “15%” sample is absent.

(b) in “Material and Methods”, page 6, “HEK293 metabolic labeling” there should be a space between “protocol was followed” and “[6]” (line 134).

(c) In “Results”, line 231, a bracket is missing in front of “A”

2. Why write “In vitro splicing was carried out …for 90 min …” if the time points were “1 hour” and “2 hours” and there is no “90 min” time point at all (in “Materials and Methods”).

3. In Figure 1A, please, mark the 3’ and 5’ splice sites in addition to just indicating their strength.

4. Is there any explanation to why the total signal is dramatically weaker at “1 hour” as compared to “t=0” input RNA specifically at 0% and 2.5% labeling (in Figure 1B)? Also, why the pre-mRNA band at t=0 is significantly weaker at 100% 4sU incorporation as compared to 0%, 2.5% and 30%? If different amount of pre-mRNA was used for in vitro splicing, please, specify in “Materials and Methods”.

5. Why “24 h” ZNF711 signal is so weak in the presence of 4sU?

6. In Figure 4B, please, provide quantitation for ADAP2 similarly as you did for DOLPP1 since visually (gel image) 4sU labelling at 24 hr appears to cause a difference in ADAP2 splicing pattern. Maybe even provide quantitation for all five exon skipping events.

Reviewer #2: The manuscript “The influence of 4-thiouridine labeling on pre-mRNA splicing outcomes” by Altieri and Hertel describes how the commonly used 4SU modified base has limited effect on splicing in cells at the concentrations employed for most experiments. This result should give other researchers confidence that use of 4SU should not strongly bias their experimental outcomes for constitutive splicing. However, it also is a reminder that the splicing events most responsive to regulation, are also most likely to be sensitive to experimental conditions. The authors also examine in vitro RNA splicing and find some changes in splicing efficiency and stability with very high incorporation of 4SU. Overall, the data are clearly presented, and their conclusions are generally well supported. The authors should address the following minor points to improve the presentation of the studies.

1- For in vitro splicing assays, the methods should explicitly describe the calculation to determine splicing efficiency, which is typically band intensity of the product mRNA divided by the summed band intensity for pre-mRNA, one of the 1st intermediate products, and mRNA- each corrected for background and the number of radiolabeled nucleotides incorporated in each species.

2-Changes in in vitro splicing efficiency can also be due to differential stability of the RNA species. Indeed, the data shown in Fig. 1B indicates that the pre-mRNA is apparently disappearing at higher rate than mRNA formation. The authors also show that high 4SU incorporation stabilizes the pre-mRNA for the AdML transcript. Is it possible that the calculated decrease in splicing with high 4SU may instead reflect increased pre-mRNA stability? Discussion of this potentially confounding issue is needed.

3-What type of variation is seen with degradation assay shown if Figure 2?

4- The nature of experimental replicates for Figures 4 and 5 should be stated. Are they biological replicates of RNA extracted from cells or technical replicates of RT-PCR analysis? The former would better support the conclusions related to 4SU induced splicing changes for the same reason that they are examining sensitive splicing events.

5- Is there a possibility that 4SU induces aberrant splicing? There are some new bands that appear at 24 hr with RSU appearing with DOLP11, TRA2B and DDX5.

********** 

6. PLOS authors have the option to publish the peer review history of their article (what does this mean?). If published, this will include your full peer review and any attached files.

If you choose “no”, your identity will remain anonymous but your review may still be made public.

**Do you want your identity to be public for this peer review?** For information about this choice, including consent withdrawal, please see our Privacy Policy.

Reviewer #1: No

Reviewer #2: No

[NOTE: If reviewer comments were submitted as an attachment file, they will be attached to this email and accessible via the submission site. Please log into your account, locate the manuscript record, and check for the action link "View Attachments". If this link does not appear, there are no attachment files.]

While revising your submission, please upload your figure files to the Preflight Analysis and Conversion Engine (PACE) digital diagnostic tool, https://pacev2.apexcovantage.com/. PACE helps ensure that figures meet PLOS requirements. To use PACE, you must first register as a user. Registration is free. Then, login and navigate to the UPLOAD tab, where you will find detailed instructions on how to use the tool. If you encounter any issues or have any questions when using PACE, please email PLOS at figures@plos.org. Please note that Supporting Information files do not need this step.

Attachment Submitted filename: Review.pdf Click here for additional data file.

# Author comment: Author response to Decision Letter 0

**DOI:** 10.1371/journal.pone.0257503.r002

22 Nov 2021

We thank the reviewers for their thorough and careful reading of the manuscript. We have addressed their comments and concerns to the best of our abilities. We have made the necessary changes to the manuscript per the reviewers’ feedback, and we have addressed the questions raised in our responses. The reviewer’s critiques are listed first and our responses are headed by the word “Response”: 

Reviewer #1:

The presented study is significant, since until now consequences of 4sU labeling on nascent pre-mRNA splicing have not been evaluated. The results of the study indicate that pre-mRNA splicing can be impacted by the incorporation of 4sU into pre-mRNA. The effect was particularly pronounced in in vitro experiments (splicing, transcription, and pre-mRNA stability) at elevated levels of 4sU incorporation. In addition, how much 4sU influenced splicing depended on the strength of the splice sites in question. Overall, the results showed that in case of splicing, the incorporation of 4sU into pre-mRNAs is not without consequences and can affect pre-mRNA splicing. The consequences become detectable at elevated levels of 4sU incorporation and in the context of suboptimal splice sites. Therefore, the study suggests that appropriate controls should be included when metabolic labeling is used.

1. Check for mistakes. For example,

(a) in “Materials and Methods” it is stated that in in vitro transcription the relative amount of 4sU was 0%, 2.5%, 15%, 30%, 100%. However, in the Results “15%” sample is absent.

Response: 

We thank the reviewer for his/her positive feedback on our work and their careful reading of our manuscript. We are sorry for the oversight mistake. In the revised manuscript we have fixed this error.

(b) in “Material and Methods”, page 6, “HEK293 metabolic labeling” there should be a space between “protocol was followed” and “[6]” (line 134).

Response:

We are sorry for the oversight mistake. In the revised manuscript we have fixed this error.

(c) In “Results”, line 231, a bracket is missing in front of “A”

Response:

We are sorry for the oversight mistake. In the revised manuscript we have fixed this error.

2. Why write “In vitro splicing was carried out …for 90 min …” if the time points were “1 hour” and “2 hours” and there is no “90 min” time point at all (in “Materials and Methods”).

Response:

Thank you for pointing out this mistake. In the revised manuscript we have fixed this error.

3. In Figure 1A, please, mark the 3’ and 5’ splice sites in addition to just indicating their strength.

Response:

The revised Figure 1 now defines the 3’ and 5’ splice sites and their strength.

4. Is there any explanation to why the total signal is dramatically weaker at “1 hour” as compared to “t=0” input RNA specifically at 0% and 2.5% labeling (in Figure 1B)? Also, why the pre-mRNA band at t=0 is significantly weaker at 100% 4sU incorporation as compared to 0%, 2.5% and 30%? If different amount of pre-mRNA was used for in vitro splicing, please, specify in “Materials and Methods”.

Response:

At optimized in vitro splicing conditions (energy source, energy regeneration source, MgCl2, DTT) T7 polymerase-generated pre-mRNA (only cap analog, no poly A tail) is attacked and degraded by RNases contained within nuclear extract. This happens within the first ~15 minutes of the splicing reaction as spliceosomal components and RNases compete for the pre-mRNA that has been added to the reaction. RNAs that associate with the spliceosome are protected from this degradation. In a typical splicing reaction ~50-75% of the input RNA is degraded quickly. This is a well-documented phenomenon that is often countered by loading different amounts of the splicing reaction onto the gel – with the result that time courses do not show the visual loss of RNA intensity. As splicing efficiencies at various time points are calculated based on the relative representation of pre-mRNA and mRNA, loading only a fraction of a time point will not alter the calculated splicing efficiency. We have opted not to load fractions of time points, but the entire reaction for each time point. As a result, our in vitro splicing gels show differential total counts in each of the time point lanes. In the revised manuscript, we state our loading approach in the ‘Materials and Methods’.

5. Why “24 h” ZNF711 signal is so weak in the presence of 4sU?

Response:

We thank the reviewer for pointing out this anomaly, which was caused by a loading error. We have re-run the experiments and now show an updated RT-PCR image. The corresponding quantitation was updated accordingly.

6. In Figure 4B, please, provide quantitation for ADAP2 similarly as you did for DOLPP1 since visually (gel image) 4sU labelling at 24 hr appears to cause a difference in ADAP2 splicing pattern. Maybe even provide quantitation for all five exon skipping events.

Response:

As suggested, in the revised manuscript all images showing RT-PCR results are now accompanied with quantitation. 

Reviewer #2:

The manuscript “The influence of 4-thiouridine labeling on pre-mRNA splicing outcomes” by Altieri and Hertel describes how the commonly used 4SU modified base has limited effect on splicing in cells at the concentrations employed for most experiments. This result should give other researchers confidence that use of 4SU should not strongly bias their experimental outcomes for constitutive splicing. However, it also is a reminder that the splicing events most responsive to regulation, are also most likely to be sensitive to experimental conditions. The authors also examine in vitro RNA splicing and find some changes in splicing efficiency and stability with very high incorporation of 4SU. Overall, the data are clearly presented, and their conclusions are generally well supported. The authors should address the following minor points to improve the presentation of the studies.

1- For in vitro splicing assays, the methods should explicitly describe the calculation to determine splicing efficiency, which is typically band intensity of the product mRNA divided by the summed band intensity for pre-mRNA, one of the 1st intermediate products, and mRNA- each corrected for background and the number of radiolabeled nucleotides incorporated in each species.

Response:

As suggested, the revised manuscript now contains a description how the efficiency of splicing was calculated. This information is included in the ‘Materials Methods’ section.

2-Changes in in vitro splicing efficiency can also be due to differential stability of the RNA species. Indeed, the data shown in Fig. 1B indicates that the pre-mRNA is apparently disappearing at higher rate than mRNA formation. The authors also show that high 4SU incorporation stabilizes the pre-mRNA for the AdML transcript. Is it possible that the calculated decrease in splicing with high 4SU may instead reflect increased pre-mRNA stability? Discussion of this potentially confounding issue is needed.

Response: 

The reviewer pointed out a plausible alternative explanation for the changes in splicing efficiency, namely that differential degradation rates of the pre-mRNA might influence calculated splicing efficiencies. To understand the influence of degradation differences on splicing differences it is best to evaluate the profiles of our �-globin substrate. It shows the most striking splicing differences when comparing fully and not modified RNA substrates (Fig. 1B,C). However, as stated in the manuscript, we did not detect statistically significant differences in the stability of the 0% and 100% 4sU modified pre-mRNAs. Thus, for the �-globin substrate the difference in splicing is not influenced by stability differences – it is all splicing. A similar analysis of the compounding splicing/degradation factors for the AdML substrate shows minimal, but not statistically significant splice differences (Fig. 1D), but stability differences (Fig. 2). Thus, it is possible that the trend of reduced splicing efficiency observed for the AdML constructs (although not statistically significant) could be a consequence of stability differences. So, for AdML, these potentially confounding effects cannot be uncoupled, as hinted to by the reviewer. In the revised manuscript now discuss the potential of these confounding factors.

3-What type of variation is seen with degradation assay shown if Figure 2?

Response:

We apologize for not showing the variation of the data shown in Figure 2. This has been corrected and the revised figure now shows that variation with error bars. We thank the reviewer for pointing out this oversight.

4- The nature of experimental replicates for Figures 4 and 5 should be stated. Are they biological replicates of RNA extracted from cells or technical replicates of RT-PCR analysis? The former would better support the conclusions related to 4SU induced splicing changes for the same reason that they are examining sensitive splicing events.

Response:

All replicates are biological replicates, and the revised manuscript now explicitly states this.

5- Is there a possibility that 4SU induces aberrant splicing? There are some new bands that appear at 24 hr with 4SU appearing with DOLP11, TRA2B and DDX5.

Response:

Yes, it is possible that 4sU induces aberrant splicing. The reviewer is correct in pointing out that additional RT-PCR bands appear in the 24hrs time point for the genes listed. Without sequencing the PCR product, it is difficult to assign identities to these bands, meaning assigning whether the bands represent aberrant splicing. In the revised manuscript we now point out the additional bands as possible, but unconfirmed evidence of aberrant splicing.

Attachment Submitted filename: Response to Reviewers.docx Click here for additional data file.

# Editor report: Decision Letter 1

**DOI:** 10.1371/journal.pone.0257503.r003

**Authors:** Ravindra N Singh

24 Nov 2021

The influence of 4-thiouridine labeling on pre-mRNA splicing outcomes

PONE-D-21-28397R1

Dear Dr. Hertel,

We’re pleased to inform you that your manuscript has been judged scientifically suitable for publication and will be formally accepted for publication once it meets all outstanding technical requirements.

Within one week, you’ll receive an e-mail detailing the required amendments. When these have been addressed, you’ll receive a formal acceptance letter and your manuscript will be scheduled for publication.

An invoice for payment will follow shortly after the formal acceptance. To ensure an efficient process, please log into Editorial Manager at http://www.editorialmanager.com/pone/, click the 'Update My Information' link at the top of the page, and double check that your user information is up-to-date. If you have any billing related questions, please contact our Author Billing department directly at authorbilling@plos.org.

If your institution or institutions have a press office, please notify them about your upcoming paper to help maximize its impact. If they’ll be preparing press materials, please inform our press team as soon as possible -- no later than 48 hours after receiving the formal acceptance. Your manuscript will remain under strict press embargo until 2 pm Eastern Time on the date of publication. For more information, please contact onepress@plos.org.

Kind regards,

Ravindra N Singh, Ph.D.

Academic Editor

PLOS ONE

Additional Editor Comments (optional):

Reviewers' comments:

# Editor report: Acceptance letter

**DOI:** 10.1371/journal.pone.0257503.r004

**Authors:** Ravindra N Singh

3 Dec 2021

PONE-D-21-28397R1 

The influence of 4-thiouridine labeling on pre-mRNA splicing outcomes 

Dear Dr. Hertel:

I'm pleased to inform you that your manuscript has been deemed suitable for publication in PLOS ONE. Congratulations! Your manuscript is now with our production department. 

If your institution or institutions have a press office, please let them know about your upcoming paper now to help maximize its impact. If they'll be preparing press materials, please inform our press team within the next 48 hours. Your manuscript will remain under strict press embargo until 2 pm Eastern Time on the date of publication. For more information please contact onepress@plos.org.

If we can help with anything else, please email us at plosone@plos.org. 

Thank you for submitting your work to PLOS ONE and supporting open access. 

Kind regards, 

PLOS ONE Editorial Office Staff

on behalf of

Dr. Ravindra N Singh 

Academic Editor

PLOS ONE
